# “Long term speech outcomes after using the Sommerlad technique for primary palatoplasty: a retrospective study in the Wilhelmina Children’s Hospital, Utrecht.”

**DOI:** 10.1007/s00784-024-05828-7

**Published:** 2024-07-24

**Authors:** Lieke Hofman, Emma C. Paes, Sarah J. Haverkamp, Kevin Jenniskens, Aebele B. Mink van der Molen

**Affiliations:** 1 Department of Pediatric Plastic Surgery, Wilhelmina Children’s Hospital, University Medical Center Utrecht, University of Utrecht, Heidelberglaan 100, Utrecht, 3508 GA the Netherlands; 2 Speech and Language Therapy, Wilhelmina Children’s Hospital, University Medical Center Utrecht, University of Utrecht, Utrecht, the Netherlands; 3 Department of Epidemiology & Health Economics, Julius Center for Health Sciences and Primary Care, University Medical Center Utrecht, Utrecht University, Utrecht, the Netherlands; 4 Cochrane Netherlands, Julius Center for Health Sciences and Primary Care, University Medical Center Utrecht, Utrecht University, Utrecht, the Netherlands

**Keywords:** Cleft lip and/Or palate, Cleft palate, Palatoplasty, Cleft palate repair, Velopharyngeal insufficiency, Speech correcting surgery

## Abstract

**Objectives:**

After cleft lip and/or palate (CL/P) repair, children may develop velopharyngeal insufficiency (VPI) leading to speech imperfections, necessitating additional speech correcting surgery. This study examines the incidence of VPI and speech correcting surgery after Sommerlad’s palatoplasty for CL/P, and its association with various clinical features.

**Materials and methods:**

A retrospective cohort study was performed in the Wilhelmina Children’s Hospital in Utrecht and child records from 380 individuals with CL/P registered from 2008 to 2017 were retrospectively reviewed. Inclusion criteria comprised the diagnosis of CL/P, primary palatoplasty according to Sommerlad’s technique, and speech assessment at five years or older. Association between cleft type and width, presence of additional genetic disorders and postoperative complications (palatal dehiscence, fistula) were assessed using odds ratios and chi squared tests.

**Results:**

A total of 239 patients were included. The VPI rate was 52.7% (*n* = 126) and in 119 patients (49.8%) a speech correcting surgery was performed. Severe cleft type, as indicated by a higher Veau classification, was associated with a significant higher rate of speech correcting surgeries (*p* = 0.033). Significantly more speech correcting surgeries were performed in patients with a cleft width >10 mm, compared to patients with a cleft width ≤10 mm (*p* < 0.001). Patients with oronasal fistula underwent significantly more speech correcting surgeries than those without fistula (*p* = 0.004). No statistically significant difference was found in the incidence of speech correcting surgery between patients with and without genetic disorders (*p* = 0.890).

**Conclusions/clinical relevance:**

Variations in cleft morphology, cleft width and complications like oronasal fistula are associated with different speech outcomes. Future research should focus on creating a multivariable prediction model for speech correcting surgery in CL/P patients.

**Supplementary Information:**

The online version contains supplementary material available at 10.1007/s00784-024-05828-7.

## Introduction

Cleft lip and/or palate (CL/P) is a congenital abnormality and often associated with additional anomalies or syndromes (in as many as 25%) [1]. The prevalence varies from 1 per 700 births worldwide to 1,5 per 1.000 births in the Netherlands [2, 3]. Care for children with a CL/P is complex and requires a multidisciplinary approach [4]. Early surgical treatment is important because children with an overt cleft may experience feeding difficulties, middle ear disease and hearing restrictions and speech disorders [5–7]. The main goal of surgical treatment is to restore the abnormal palatal anatomy to improve velopharyngeal function and speech.

Current surgical treatment protocols exhibit a significant degree of heterogeneity, lacking a global consensus among surgeons [8, 9]. Yet, all techniques aim to close the cleft and obtain normal speech development and undisturbed maxillofacial growth. A frequently employed repair technique is Sommerlad’s straight-line palatoplasty [10]. This approach involves linear closure of the soft palate and intravelar veloplasty to reposition the velum muscles posteriorly. Repair of the hard palate may be accomplished using a vomer flap, with the intention of enhancing maxillary growth through the reduction of denuded palatal bone area and resultant minimization of scarring [11].

After palatoplasty, children may develop velopharyngeal insufficiency (VPI) with speech imperfections necessitating speech therapy and/or additional surgical interventions, such as speech correcting surgery [12]. VPI is defined as the incomplete separation of oral and nasal cavities during speech and manifests up to 47% of children with a cleft palate [13]. Speech problems are usually related to resting VPI. A recently performed literature study showed a high heterogeneity in both surgical techniques and speech outcomes. Most speech correcting surgeries were performed after straight-line palatoplasty when compared to Z-palatoplasty and palatoplasty with adding buccal flaps. The diagnosis and rating of VPI remains a complex process, requiring coordinated care by surgeons and speech-language pathologists (SLPs). The etiology of VPI is multifactorial and may depend on the type and success of primary palatoplasty. The outcome of a primary palatoplasty may be influenced by patient specific factors, including cleft type and width, timing of repair, genetic disorders, surgeon skills and postoperative complications [14–16].

The cleft team of the Wilhelmina Children’s Hospital in Utrecht, the Netherlands, changed their treatment protocol in 2008 according to the Sommerlad technique. The rationale was to improve speech outcomes by closing the whole palate in the first year of life, with the least impact on growth. In the former protocol, soft palate closure was performed using intravelar veloplasty by Perko and the hard palate was closed at later stadium using the Von Langenbeck technique [17]. The primary goal of the old protocol was to achieve optimal growth by postponing hard palate closure to the age of three or four years. However, a later retrospective study of patients treated by the old protocol showed only moderate good growth results after closure of the hard palate around at later age [17]. Also, previous studies have shown contradictory outcomes regarding speech after delayed hard palate closure. This study aims to evaluate the incidence of VPI and the need for speech correcting surgery after Sommerlad’s technique for CL/P, and to explore the association with various clinical features. We seek to investigate the outcome after switching treatment protocol to assess its efficacy and thereby aim to contribute to enhancing surgical care. Besides, we aim to address the gap in the existing literature on the influence on different variables on speech outcome after palatoplasty.

## Materials and methods

### Clinical data and demographics

This retrospective cohort study was performed in the Wilhelmina Children’s Hospital in Utrecht, the Netherlands, and approved by our institutional review board. Child records from individuals registered from 2008 to 2017 were retrospectively reviewed. All children underwent standardized assessments according to a predetermined treatment protocol. Patients were included if they had a cleft palate, underwent Sommerlad palatoplasty, were aged five years or older and when speech assessment by the SLP at the time of query was done. Exclusion criteria were submucous cleft palate (SMCP), adoption, primary surgery in another hospital, loss to follow-up or lack of medical records. Patients’ charts were accessed through the hospital’s medical database and information regarding date of birth, gender, genetic disorder, cleft morphology, cleft width, age at palatal repair, palatal wound dehiscence, oronasal fistula, speech assessment, and need for speech correcting surgery were extracted.

### Surgical procedures

All included patients underwent straight-line palatoplasty with intravelar veloplasty (IVV) as described by Sommerlad [10]. The surgeries were performed by two experienced cleft surgeons. In a complete palatal cleft, hard and soft palate closure was performed in one procedure (vomerplasty and straight-line soft palate closure with IVV). In case of a complete CL/P, the hard palate was closed with a vomerine flap simultaneous with lip closure and closure of the soft palate with IVV was performed 3–6 months later.

The surgery was performed under general anesthesia, with a suprazygomatic nerve block. Prophylactic Cefazoline (30 mg/kg) were administered intravenously. The palate was infiltrated with lidocaine 2% and adrenaline 1:80.000 for hemostatic reasons. The procedure was performed under the operative microscope. The hard palate is closed by incising the mucosa from the vomer on one side of the cleft to raise a vomer flap. Subsequently, the oral mucosa is incised at the cleft margin. The vomer flap is then fixated between the border of the hard palate and the oral mucosa. For closure of the soft palate, the oral mucosa is incised along the margins of the cleft at the junction between the oral and nasal mucosa and extended onto the posterior hard palate. The incision is then extended backwards and laterally to mobilize the muscles and nasal mucosa from the posterior border of the hard palate. The oral mucosa is then carefully mobilized from the muscles of the velum and the nasal mucosa and any attachments to the posterior edge of the hard palate. Next, the nasal mucosa is sutured in the midline to provide tension necessary for further muscle dissection. The levator veli palatini muscles are dissected out and freed from any mucosal connections far laterally up into the levator tunnel and checked for good excursion, retropositioned and fixated in the midline to create the new muscular sling. Finally, the oral mucosa is sutured in the midline. To occlude dead space and to keep the repaired levator muscle sling in its posterior position, quilting mattress sutures between the oral and nasal mucosa are performed anteriorly of the muscle. If there is too much tension on the oral mucosa, uni- or bilateral relaxing incisions are made.

### Speech assessment

Cleft speech assessments were completed at annual cleft team visits by one the three SLP’s of our team (with > 15 years of experience) according to the Dutch Cleft Speech Evaluation Test (DCSET). The minimum postoperative time for speech assessment in our center is at age three and according to the protocol speech assessment was performed at age three and age five. The reliability of the DCSET has been studied by Spruijt et al. (2017), the strength of intra- and interrater agreement for most of the parameters is good or very good [18]. For this study, the available clinical ratings were used. The SLP’s of our center have ample cleft experience and do participate in the mandatory national calibration sessions of all Dutch Cleft SLP’s twice yearly. No independent re-assessment was done and therefore the inter- and intra-rater reliability have not been tested. For this study, patients’ resonance, nasal air emission, and scored intelligibility was used from the 5 years speech assessment. Resonance was scored on a 4-point scale. The nasal mirror test was used to detect nasal air emission and was scored as present or absent. Speech intelligibility was scored on a 5-point scale during spontaneous speech by both the parents and the SLP (**Online Resource**** 1**).

### Data collection

The postoperative outcomes such as VPI, speech correcting surgery, palatal wound dehiscence and oronasal fistula, were recorded. The primary outcome of this study is VPI after primary palatoplasty. Speech variables such as hypernasality, nasal air emission and poor intelligibility are indicators for VPI. Additional speech correcting surgery was offered to patients and parents when the existence of VPI was agreed upon members of the cleft team and further speech therapy was considered to be ineffective. Palatal wound dehiscence was defined as a one-layer opening in the palate with spontaneous closure in the first 3 months following surgery. Oronasal fistula was defined as a persistent two-layer opening following three months after primary palatoplasty. Both diagnoses were confirmed by a plastic surgeon.

Individual characteristics associated with the subsequent need for speech correcting surgery were scored as secondary outcomes: age of primary palatoplasty, cleft type, cleft width, genetic disorder, palatal wound dehiscence, and oronasal fistula. Cleft characteristics were distinguished and classified according to the Veau classification (Veau I, soft cleft palate; Veau II, soft and hard cleft palate; Veau III, unilateral cleft lip, alveolus and palate, Veau IV, bilateral cleft lip, alveolus and palate). Cleft width was measured at the junction of the hard and soft palate (in mm), before and after vomerplasty. Genetic disorders were defined as diagnosis of a syndrome by geneticists or confirmed through positive genetic testing for mutations associated with palatogenesis. Non-syndromic Robin Sequence (RS) was distinctly classified, determined both by geneticists’ diagnosis and by physical examination conducted by the plastic surgeon.

### Data analysis

For statistical analysis, IBM SPSS software, version 26 was used. Results were presented as means with standard deviations and percentages. Odds ratios (OR) and 95% Confidence Intervals (CI) were calculated. Association between variables and the incidence of speech correcting surgery was analyzed using the chi squared (X^2^)test for categorical variables. Variables consisting of more than two groups were also assessed using X^2^ tests. A p-value < 0.05 was regarded as statistically significant.

## Results

A total of 380 records were retrieved and assessed for eligibility. Two hundred thirty-nine patients were included for data analysis (**Online Resource**** 2**). Mean duration of follow-up was 10 years (range 5–16 years, SD 3.0). Patient characteristics are summarized in Table 1.

**Table 1:** Patient characteristics

Patient characteristics	Patients *n* = 239	Patients %
Gender Female Male	111 128	46.4 53.6
Genetic disorder Yes Non-syndromic RS	62 24	25.9 10.0
Mean age at soft palatoplasty	10 months (range 3–22 months, SD 2.8)	
Cleft morphology Veau I Veau II Veau III Veau IV	33 85 90 31	13.8 35.6 37.7 13.0
Mean primary cleft width	10.9 mm (range 3–22, SD 3.3)	
Primary cleft width ≤10 mm >10 mm	90 128	37.7 53.6

*Cleft width was measured at the junction between the hard and soft palate*,* before the first palatoplasty. Abbreviations: RS*,* Robin Sequence*

### Speech outcome

Mean age at speech assessment was 5 years (SD 1.34). After primary palatoplasty, normal resonance was observed in 32.6% (*n* = 78), mild hypernasal resonance in 25.1% (*n* = 60), moderate hypernasal resonance in 23.8% (*n* = 57) and severe hypernasal resonance in 16.7% (*n* = 40). Nasal air emission, based on nasal mirror test, was detected in 66.5% (*n* = 159) and absent in 20.5% (*n* = 49) of the patients. The average speech intelligibility, as reported by parents, was 2.2 (ranging from 1 to 5, SD 0.97), and 2.6 (ranging from 1 to 5, SD 1.01) as reported by SLP (**Online Resource**** 3**). The rate of VPI was 52.7% and in 119 patients (49.8%) a secondary speech correcting surgery was performed.

### Age of first surgery

Mean age at soft palatoplasty was 10 months (range 3–22 months, SD 2.8). One patient underwent delayed soft palatoplasty (22 months) due to cardiac issues necessitating cardiac surgery (with no requirement for speech correction surgery). No significant difference regarding incidence of speech correcting surgery was found between patients who underwent soft palatoplasty before and after 10 months of age (*p* = 0.272) (Table 2).

**Table 2:** Effect of different variables on speech correcting surgery rate

	Speech correcting surgery (%)	*p* ^a^
Age ≤10 months of age >10 months of age	61 (46.6) 58 (53.7)	0.272
Veau classification Veau I Veau II Veau III Veau IV	11 (33.3) 37 (43.5) 53 (58.9) 18 (58.1)	0.033
Cleft width ≤10 mm >10 mm	30 (32.3) 76 (60.8)	< 0.001
Genetic disorder Yes No RS without genetic disorder	30 (48.4) 76 (49.7) 13 (54.2)	0.890
Date of surgery Surgery between 2007–2010 Surgery between 2015–2018	37 (51.4) 31 (39.7)	0.152

^*a*^ *P-value based on chi squared test*

### Cleft morphology

Majority of patients had a Veau III type of cleft (*n* = 90; 37.7%). Cleft severity showed a significant association with subsequent need for speech correcting surgery (*p* = 0.033) (Table 2). The odds of undergoing speech correcting surgery were 1,8 times higher for Veau I compared with Veau III (OR 1,753, *p* = 0.014) and 1,7 times higher compared with Veau IV (OR 1,742, *p* = 0,047) and 1,4 times higher for Veau II compared with Veau III (OR 1,353, *p* = 0,042) (Table 3).

**Table 3:** Odds ratios (OR), confidence intervals (CI) and significance results (p) for speech correcting surgery rate with cleft type

	Speech correcting surgery		
	OR	95% CI	*p* ^a^
Cleft type Veau I vs. Veau II Veau I vs. Veau III Veau I vs. Veau IV Veau II vs. Veau III Veau II vs. Veau IV Veau III vs. Veau IV	1,306 1,753 1,742 1,353 1,334 0,986	0,761–2,241 1,049–2,929 0,987–3.073 1,005–1,821 0,908–1,960 0,698–1,393	0,312 0,014 0,047 0,042 0,165 0,936

^a^ P-value based on chi squared test

### Cleft width

Cleft width before primary palatoplasty was reported in 218 patients (91.2%). Mean cleft width was 10.9 mm (range 3–22 mm, SD 3.3). Ninety patients (41.3%) had a cleft width ≤10 mm and 128 patients (58.7%) >10 mm (range 11–22). Patients with a cleft width >10 mm underwent significant more speech correcting surgeries (60.8%) compared to ≤10 mm (32.3%) (*p* < 0.001) **(**Table 2**).**

### Genetic disorder

Sixty two patients (25.9%) were included with genetic disorders and 24 patients (10.0%) with RS (without genetic disorder) (**Online Resource**** 4**>). The difference between patients with and without genetic disorders regarding incidence of speech correcting surgery was not significant (*p* = 0.890) (Table 2).

### Surgeon skills

Surgeries were performed by two surgeons. No differences in postoperative complications, VPI and speech correcting surgery rate were seen between them. During the first four years of the study period (51.4%), more patients underwent speech correcting surgery, when compared to the final four years (2015–2018) (39.7%), but not significant (*p* = 0.152) (Table 2).

### Postoperative complications

Ten percent (*n* = 24) was diagnosed with palatal wound dehiscence. Five did not heal spontaneously within 3 months and were thus classified as oronasal fistula. After 3 months of surgery, 18% (*n* = 43) had a persistent two-layer oronasal fistula, the majority were functional. Eighty-six percent (*n* = 37) of patients with fistula underwent surgery to address the fistula. No significant difference in the need for speech correcting surgery was observed in patients with and without palatal wound dehiscence (*p* = 0.378). Patients with fistula underwent significant more speech correcting surgeries (*p* = 0.004) **(**Table 4**).**

**Table 4:** Effect of postoperative complications on speech correcting surgery rate

	Speech correcting surgery (%)	*p* ^a^
Palatal dehiscence Yes No	14 (58.3) 105 (48.8)	0.378
Fistula Yes No	30 (69.8) 89 (45.4)	0.004

^*a*^ *P-value based on chi squared test*

## Discussion

In this retrospective study, long-term speech outcomes following Sommerlad straight-line palatoplasty were evaluated over a period of 10 years. The incidence of VPI and speech correcting surgery are 52.7% and 49.8%, respectively.

Our results are leveling the upper range when compared to the literature [13]. They align with Mapar et al. (2019), reporting a VPI rate of 42.5% after Sommerlad palatoplasty [16]. However, they contrast Baillie & Sell (2020), reporting a VPI rate of 5.2% after Sommerlad palatoplasty, performed by Sommerlad himself [15]. In general, between studies, there is a substantial heterogeneity in terms of study methodologies, inclusion and exclusion criteria, treatment protocols and reported patient characteristics making national or international comparison inequitable. No exact definition or threshold for VPI is confirmed and there is a lack of clear criteria for VPI between centers with high variability in speech assessments. Also, cleft teams throughout the world show cultural differences regarding the subjective element of speech, shared decision making, and different thresholds to re-operate. Little is known about the possible variation in the effect of some VPI on the understandability of speech in different languages. In our institution, we aim for good clear speech without signs of VPI for any cleft patient. We hypothesize that our cleft team may maintain a lower threshold to reoperate in comparison to other institutes, potentially leading to higher reoperation rates. While emphasizing the importance of shared decision making in our hospital, we advocate for the guided facilitation of this process, often recommending additional surgery if the child’s speech has signs of VPI. Standardized assessment criteria are lacking, necessitating the development universally accepted protocols [13].

### Surgical technique

In the literature, Sommerlad palatoplasty have shown excellent results [15]. Though, some studies reported better speech outcomes following Furlow Z-palatoplasty compared to straight-line palatoplasty [19]. The Z-palatoplasty technique is associated with less linear contractile forces secondary to scar formation, as the arms of the Z interrupt the linear scar, decreasing the amount of secondary shortening of the soft palate. Despite better speech outcomes after Z-palatoplasty, challenges may arise in closing wider clefts [20]. To address this difficulty, additional buccal flaps may be implemented [21].

### Correlation between cleft width, cleft type and incidence of fistula and speech correcting surgery

Prior studies demonstrated that patients with complete CL/P generally manifest wider and shorter clefts when compared to patients with isolated cleft palates. Those studies also demonstrated that wider clefts are associated with higher Veau classifications [22]. This agrees with our results, showing significant more patients with cleft width ≤10 mm in Veau class I and II, and significant more patients with cleft width >10 mm in higher Veau classes. These observations underscore the value of considering cleft width and type as essential factors in clinical decision-making, potentially informing personalized treatment strategies for patients. Besides, this study demonstrates that two-stage surgery with hard palate closure using vomerplasty during first surgery significantly decreases the soft cleft width with 4.2 mm (95% confidence interval [CI] = 3.6–4.7) (*p* < 0.001) at the time of soft palate closure.

Patients with wider clefts are more prone to develop palatal dehiscence and oronasal fistula, which may increase the chance to develop VPI and suboptimal speech outcomes [23]. Our data showed a higher rate of palatal wound dehiscence and fistulae in patients with wider (>10 mm) and more extensive cleft types (higher Veau classifications). Oronasal fistula serve as significant predictor for the onset of VPI [24]. Our study affirms this observation, indicating that patients with oronasal fistulae are more prone to undergo additional speech correcting surgeries in comparison to their counterparts without complications. The incidence of oronasal fistulae in our study falls within the range reported in other publications over the past decade [25–27].

### Genetic disorder

Additional challenges and considerations arise in treatment protocols for patients with genetic disorders. Despite these, studies show comparable VPI rates, suggesting other influential variables in predicting speech outcomes [28]. Hardwicke et al. (2016), conducted a comparative analysis of speech outcomes in CL/P patients with genetic disorders and/or malformations and those with isolated cleft palate [29]. Although higher VPI rates were found in RS patients, results were matched for gender, age at repair, and cleft severity. Consequently, researchers concluded that factors intrinsic to RS may contribute to less favorable speech outcomes.

We found no significant difference in speech outcomes between cleft palate patients with and without genetic disorders. This suggests that other variables, not limited to genetic disorder, exert a more leading role on speech outcomes after primary palatoplasty. Furthermore, a discrepancy between cleft width and the presence of genetic disorders was observed. Wider clefts were found in patients without a genetic disorder when compared to patients with a genetic disorder or non-syndromic RS (*p* < 0.001), contradicting previous findings [26, 28, 30, 31]. To our knowledge, no studies have been exclusively dedicated to investigating differences between cleft width in patients with and without genetic disorders and/or other malformations [32].

### Surgeon skills

The surgeons’ skill is also a variable that warrants consideration in any surgical outcome, including speech outcomes after palatoplasty. However, the influence of the surgeon’s experiences and surgical skills are sparsely discussed in the literature [33]. This study revealed no differences in speech outcomes between the two surgeons involved in performing the surgeries. We observed better speech outcomes in patients who underwent surgery during final four years of the study period (2015–2018). The learning curve, performing a more radical muscle dissection of the levator muscle during the years, and the constant drive to improve results may play a role, even though our surgeons were experienced.

### Limitations

Due to the retrospective design of the study, studying medical records might led to inaccuracy, is subject to confounding errors and, data of some patients was missing of patients were lost to follow-up. Despite, the determination of the inter- and intra-rater reliability between speech-language pathologists was not feasible within the scope of this study, the potential sources of confounding in the subjective speech assessment were mitigated by employing three experienced cleft SLP’s.

## Conclusion

In conclusion, the results demonstrate that while using the Sommerlad technique for cleft palate closure, cleft morphology and width significantly impact the need for secondary speech correcting surgery. Postoperative complications, such as fistulae, also correlate with increased chance to develop VPI and the need for speech correcting surgery. These findings emphasize the importance of considering cleft width and severity in surgical planning to improve long-term speech outcomes in CL/P patients. Future prospective research should continue to explore these associations to enhance the quality of care and speech outcomes for individuals with cleft palates.

### Electronic supplementary material

Below is the link to the electronic supplementary material.

Supplementary Material 1

Supplementary Material 2

Supplementary Material 3

Supplementary Material 4

## Data Availability

Data is provided within the manuscript or supplementary information files.
