# Supplementary material for: “Long term speech outcomes after using the Sommerlad technique for primary palatoplasty: a retrospective study in the Wilhelmina Children’s Hospital, Utrecht.”
Source: Clin Oral Investig. 2024 Jul 24;28(8):441. doi: 10.1007/s00784-024-05828-7 (PMC11269319; doi:10.1007/s00784-024-05828-7)
Supplement: Supplementary file 1 — Supplementary Material 1 [file 784_2024_5828_MOESM1_ESM.docx]

| Table, Online resource 3. Speech outcome | | | |  |  |
| --- | --- | --- | --- | --- | --- |
|  | | Patients  n=239 | % | Speech correcting surgery  (n) | % |
| Resonance | 0=Normal resonance | 78 | 32.6 | 2 | 2.6 |
|  | 1=Mild hypernasal resonance | 60 | 25.1 | 23 | 38.3 |
|  | 2=Moderate hypernasal resonance | 57 | 23.8 | 52 | 91.2 |
|  | 3=Severe hypernasal resonance | 40 | 16.7 | 40 | 100 |
| Nasal air emission | Yes (one or two nostrils) | 159 | 66.5 | 102 | 64.2 |
|  | No | 49 | 20.5 | 1 | 2.0 |
| Intelligibility (parents) | 1=The speech is understandable and normal. | 53 | 22.2 | 7 | 13.2 |
|  | 2=The speech differs from others. This does not lead to comments and the speech is understandable. | 88 | 36.8 | 37 | 42.0 |
|  | 3=The speech differs from others. This does lead to comments and the speech is understandable. | 44 | 18.4 | 33 | 75.0 |
|  | 4=The speech is understandable with some difficulty. | 23 | 9.6 | 22 | 95.7 |
|  | 5=The speech is not understandable. | 2 | 0.8 | 2 | 100 |
| Intelligibility  (SLP) | 1=The speech is understandable and normal. | 22 | 9.2 | 0 | 0 |
|  | 2=The speech differs from others. This does not lead to comments and the speech is understandable. | 106 | 44.4 | 31 | 29.2 |
|  | 3=The speech differs from others. This does lead to comments and the speech is understandable. | 55 | 23.0 | 38 | 69.1 |
|  | 4=The speech is understandable with some difficulty. | 38 | 15.9 | 36 | 94.7 |
|  | 5=The speech is not understandable. | 10 | 4.2 | 10 | 100 |

*Abbreviations: SLP, speech-language pathologist*
