# Supplementary material for: “Long term speech outcomes after using the Sommerlad technique for primary palatoplasty: a retrospective study in the Wilhelmina Children’s Hospital, Utrecht.”
Source: Clin Oral Investig. 2024 Jul 24;28(8):441. doi: 10.1007/s00784-024-05828-7 (PMC11269319; doi:10.1007/s00784-024-05828-7)
Supplement: Supplementary file 2 — Supplementary Material 2 [file 784_2024_5828_MOESM2_ESM.docx]

| Table, Online Resource 2. Excluded patients | | |  |
| --- | --- | --- | --- |
|  | | N |  |
| Total | | 380 |  |
| Exclusion | Adopted | 37 |  |
|  | SMCP or bifid uvula | 33 |  |
|  | Primary surgery in other hospital | 4 |  |
|  | Surgery according to old protocol | 19 |  |
|  | Incapable to communicate through verbal communication (in case of genetic disorder) | 7 |  |
|  | Loss to follow-up (including lack of medical records) | 41 |  |
| Inclusion |  | 239 |  |
