# Supplementary material for: “Long term speech outcomes after using the Sommerlad technique for primary palatoplasty: a retrospective study in the Wilhelmina Children’s Hospital, Utrecht.”
Source: Clin Oral Investig. 2024 Jul 24;28(8):441. doi: 10.1007/s00784-024-05828-7 (PMC11269319; doi:10.1007/s00784-024-05828-7)
Supplement: Supplementary file 3 — Supplementary Material 3 [file 784_2024_5828_MOESM3_ESM.docx]

| **Table, Online Resource 4.** Genetic disorders | |
| --- | --- |
| Type of genetic disorder | Number of patients |
| Stickler syndrome | 10 |
| Stickler syndrome + Robin Sequence | 6 |
| Van der Woude syndrome | 4 |
| Kabuki syndrome | 3 |
| Velocardiofacial (22q11.2) syndrome | 3 |
| Down syndrome | 2 |
| CHARGE syndrome | 2 |
| Mutation in SIX3 gene | 2 |
| Goldenhar syndrome | 2 |
| Sotos syndrome | 1 |
| Klinefelter syndrome | 1 |
| Beckwith-Wiedemann syndrome | 1 |
| Gorlin syndrome | 1 |
| Axenfeld-Rieger syndrome | 1 |
| Blepharo-cheilo-dontic (BCD) syndrome | 1 |
| Swachman-Diamond syndrome | 1 |
| Myhre syndrome | 1 |
| Oculo-auriculo-frontonasal (OAFNS) syndrome | 1 |
| Mosaic trisomy 10 | 1 |
| Smtih-Lemli-Opitz (SLO) syndrome | 1 |
| Amniotic Band syndrome | 1 |
| Other associated malformation or genetic mutations | 15 |
