# Supplementary material for: “Long term speech outcomes after using the Sommerlad technique for primary palatoplasty: a retrospective study in the Wilhelmina Children’s Hospital, Utrecht.”
Source: Clin Oral Investig. 2024 Jul 24;28(8):441. doi: 10.1007/s00784-024-05828-7 (PMC11269319; doi:10.1007/s00784-024-05828-7)
Supplement: Supplementary file 4 — Supplementary Material 4 [file 784_2024_5828_MOESM4_ESM.docx]

| **Table, Online Recourse 1.** Speech assessment | |
| --- | --- |
| Resonance | 0=normal resonance |
|  | 1=mild hypernasal resonance |
|  | 2=moderate hypernasal resonance |
|  | 3=severe hypernasal resonance |
|  | |
| Speech intelligibility | 1=normal and understandable speech |
|  | 2=speech that differs from others but did not lead to comments and the speech is understandable |
|  | 3=speech that differs from others and does lead to comments, but the speech is understandable |
|  | 4=understandable with some difficulty speech |
|  | 5=speech that is not understandable |
